# Empowering Preventive Care with *GECA* Chatbot

**DOI:** 10.3390/healthcare11182532

**Published:** 2023-09-13

**Authors:** Eva Maia, Pedro Vieira, Isabel Praça

**Affiliations:** GECAD—Research Group on Intelligent Engineering and Computing for Advanced Innovation and Development, School of Engineering of the Polytechnic of Porto (ISEP), 4249-015 Porto, Portugal

**Keywords:** chatbot, preventive care, COVID-19, dementia, artificial intelligence

## Abstract

Chatbots have become increasingly popular in the healthcare industry. In the area of preventive care, chatbots can provide personalized and timely solutions that aid individuals in maintaining their well-being and forestalling the development of chronic conditions. This paper presents *GECA*, a chatbot designed specifically for preventive care, that offers information, advice, and monitoring to patients who are undergoing home treatment, providing a cost-effective, personalized, and engaging solution. Moreover, its adaptable architecture enables extension to other diseases and conditions seamlessly. The chatbot’s bilingual capabilities enhance accessibility for a wider range of users, including those with reading or writing difficulties, thereby improving the overall user experience. *GECA*’s ability to connect with external resources offers a higher degree of personalization, which is a crucial aspect in engaging users effectively. The integration of standards and security protocols in these connections allows patient privacy, security and smooth adaptation to emerging healthcare information sources. *GECA* has demonstrated a remarkable level of accuracy and precision in its interactions with the diverse features, boasting an impressive 97% success rate in delivering accurate responses. Presently, preparations are underway for a pilot project at a Portuguese hospital that will conduct exhaustive testing and evaluate *GECA*, encompassing aspects such as its effectiveness, efficiency, quality, goal achievability, and user satisfaction.

## 1. Introduction

Chatbots are natural language processing systems that act as a virtual conversational agents mimicking human interactions. In recent years, the interest in chatbots is increasing, and they are being adopted by companies in several sectors, such as real estate, travel, education, healthcare, and finance [1]. More generally, chatbots such as Amazon Alexa, Google Assistant, Apple Siri and more recently ChatGPT have become popular among people, since they can support them to complete their daily routine tasks efficiently.

The growing interest in this kind of technology, allied with the need to support patients at home, triggered the development of chatbots in the healthcare sector. Using either rule-based or artificial intelligence (AI) methods, health chatbots can help to improve and automate services in the healthcare sector: namely, they can potentially improve the access to healthcare, improve doctor–patient and clinic–patient communication, or help to manage the increasing demand for health services such as via remote testing, medication adherence monitoring or teleconsultations [2]. Moreover, the COVID-19 global pandemic has underscored the necessity for healthcare services to be delivered in a home-based setting without compromising the safety of healthcare professionals or patients. Additionally, the pandemic has emphasized the urgency of developing new services that are both fast and tailored to the specific needs of the emerging situation. Also, the aging population and the rise of chronic illness, which cannot be treated at care facilities, demand a functional preventive care and treatment for these diseases at home. Monitoring patients at home, allowing them to follow medical prevention prescriptions, will help elderly people and people with chronic diseases stay healthy. This preventive health model will help prevent disease, detect disease early, and improve healthcare results.

Large Language Models (LLMs) have shown promising advancements in natural language processing, making them increasingly relevant for healthcare applications, particularly in the context of conversational AI. Some LLMs were already developed to create models with scientific domain-specific knowledge, such as Galactica [3] or BioGPT [4]. Nevertheless, few LLMS exist that used real-world data, such as EHR, due to the sensitivity of medical data. Two approaches developed include an accurate model, named BEHRT, used for the prediction of next/future diseases [5], and the GatorTron model that allows the de-identification of clinical notes [6]. Despite these promising applications of LLMs in healthcare, they appear ill-prepared to be deployed directly as patient tools, particularly as chatbots. This is due to the essential need for ensuring patient safety and accuracy beyond merely generating eloquent text outputs and human-like interactivity. LLMs may not be suitable in this context due to risks relating to the associations and biases of its training data. Therefore, domain-specific chatbots are more appropriate in such cases, offering tailored and focused interactions with users [7].

In this context, this work presents *GECA*, which is a chatbot that is dedicated to preventive care and provides patients who are undergoing home treatment with information, guidance, and monitoring. *GECA* is capable of detecting and notifying healthcare providers of any abnormal health indicators. As far as we are aware, this is the first chatbot that has been specifically designed for patients undergoing home treatment for a particular condition with an emphasis on preventive care. By utilizing this monitoring approach, patients’ recovery and health status can be constantly monitored, thereby providing support for remote testing, medication adherence, and overall health. Furthermore, if the patient requires any form of assistance, all the relevant information can be communicated to healthcare professionals. *GECA* is part of the iCare4NextG international platform [8], which provides wellness and care services that are aligned with the concepts of prediction, prevention, customization and participation, integrated care and home care. Being part of this healthcare ecosystem enhances *GECA*’s impact and effectiveness, as it collaborates within a comprehensive framework designed to serve the needs of users effectively. Initially, *GECA* will intelligently support remotely patients with COVID-19 and dementia.

The main contributions of this paper include the following:

- A thorough examination of existing healthcare chatbots documented in the literature;
- The introduction of the *GECA* chatbot, designed to address the gap in preventive care chatbots, specifically for patients undergoing home treatment for particular conditions;
- Elaboration on the architecture and implementation of the *GECA* chatbot, including the provision of interaction examples and detailed insights;
- A comprehensive discussion on the validation of *GECA*, shedding light on the challenges associated with validating healthcare chatbots, primarily due to the absence of standardized protocols.

The structure of this paper is as follows: Section 2 provides an overview of existing chatbots in the literature; Section 3 describes the architecture and implementation of *GECA* chatbot; and Section 4 summarizes the main findings and outlines future work.

## 2. Background

The idea of developing machines that can communicate with people started several decades ago. However, over time, the development of more powerful computational tools and the refinement of Natural Language Processing (NLP) techniques allowed these machines, including the chatbots, to become increasingly sophisticated in their ability to communicate effectively with users [9]. This happened in several fields, and healthcare was not an exception.

The first known chatbot used in healthcare was ELIZA [10], which was programmed in 1966 and functioned as a therapist by responding to user input with follow-up questions. ELIZA utilized the Pattern Matching and Replacement methodology to create the illusion of understanding. Another prominent chatbot, Parry [11], was created in 1972 to simulate a paranoid schizophrenic patient for the purpose of training psychiatrists. At a conference that same year, a demonstration featured a conversation between ELIZA and Parry [11]. Although ELIZA had limited knowledge and communication skills, advances in AI techniques have led to improved performance in more recent chatbots.

Chatbots have already found numerous applications in the healthcare industry. Chatbots that provide medical information, such as Healthily [12] and Ada Health [13], are some of the most widely used. Additionally, chatbots like Woebot [14] have been developed specifically to assist with mental health concerns and have gained significant popularity. These chatbots can offer cognitive behavioral therapy for conditions such as depression, post-traumatic stress disorder, and anxiety as well as help patients with autism improve their social skills. Some chatbots have also been designed to automate administrative tasks within healthcare systems, such as scheduling medical appointments. These chatbots can be integrated into the medical system and accessed through a user’s device to find a suitable physician and appointment slot. Other chatbots are designed to collect patient data by asking simple questions about personal information and symptoms, which can then be used for admission, symptom tracking, doctor–patient communication, and medical record keeping. Chatbots can even assist patients with requesting prescription refills, especially for chronic diseases that do not require medical intervention.

In order to provide an overview of chatbots developed for the healthcare industry, we have categorized them based on their intended purpose. The selected chatbots are primarily designed to provide medical information and health assistance with a focus on those that offer diagnosis, patient support (including mental health counseling), and health promotion. Furthermore, context-specific chatbots that are tailored to address the unique challenges and needs associated with major health crises and leading causes of death, such as COVID-19, cancer, and dementia have been identified. Table 1 presents a summary of the chatbots selected.

### 2.1. Chatbots for Diagnosis

Babylon, Ada Health, Buoy Health and Your.md are all chatbots that enable users to input their symptoms and receive helpful diagnoses [35]. Babylon functions by cross-referencing the symptoms provided by the user with a database of multiple diseases. This enables the user to obtain a diagnosis and make informed decisions based on the information provided. Additionally, it allows a direct interaction with a real doctor, which is not typically found in chatbots. In 2017, the United Kingdom’s National Health Service (NHS) started a trial of using this chatbot, and today, it provided over 700,000 online consultations with doctors [15]. Ada Health enables users to input their symptoms and then search for corresponding diseases, displaying the most probable matches [16]. Buoy Health is very similar to the previous chatbots. It analyzes various diseases and medical causes to propose a treatment [17]. Your.md is also focused on making diagnosis. In 2020, Aleksandar Ćirković [18] compared these four chatbots and observed that Ada Health and Your.MD showed the best results. Nevertheless, the treatment recommendations given by the chatbots for the same starting symptoms varied significantly, with Ada generally recommending emergency care for almost every diagnosis. Moreover, Ada Health asks redundant questions at the end of the consultation, such as asking about a symptom that was already mentioned earlier. Also, the additional information it provides to the users in the form of a visual description of how many patients with similar symptoms have been diagnosed with the suggested diagnosis may not be entirely accurate. Your.MD provided the most valid recommendations in the study but also raised some concerns on the dry eyes diagnosis, unnecessarily transitioning from self-treatment to emergency care.

Gyant [19] is another chatbot that aims to diagnosis and treat non-urgent medical conditions by asking the user questions about their symptoms and overall well-being. This information is also used to make recommendations to the healthcare professional, who will write the medical prescription [36]. An important feature of the Gyant chatbot is its human-like behavior due to its sense of humor, use of emojis and memes. Nevertheless, when trying to access Gyant, it is no longer available. Symptomate chatbot [20] allows its users to report their symptoms and to answer some questions about their health status to receive a list of the most likely diagnosis. The accuracy of the Symptomate chatbot was around 66%, which is a low result compared to, for example, ADA Health, which scored 80% [37]. MedWhat [21] is another chatbot that is useful to facilitate the medical diagnosis both for patients and doctors. It excels in speed, ease and transparency.

#### Well-Being Chatbots

Iona Mind [22] is an AI application which acts as a guide for “fitness of the mind”. It is a self-help application that promotes personalized plans and goals related to mental well-being, like managing stress and anxiety, to help users achieve their mental wellness goals. Despite not being designed to treat psychiatric disorders, 86% of Iona Mind users report feeling better after their first session. The Florence Nightingale Chatbot [23] aims to help English speakers manage their health and well-being, reminding them to take their medicines and helping them find health specialists. Florence also successfully tracks patients’ health to help them reach their goals. For example, it can track user’s body weight, mood or period.

Izzy [24] is designed to help menstruating people monitor their period through Facebook Messenger. Izzy can assist the user with information such as when to take birth control pills, when their fertility period is, and when their next period is due. Furthermore, Izzy can remember and identify the fertile periods and inform about sexual problems [38]. SafeDrugBot [25] provides information about the safety of a drug to breastfeeding women. It is useful for medical professionals who seek information about a specific medication and want to know if it is recommended for breastfeeding women [39].

### 2.2. Mental Health Chatbots

Elomia [26] is a chatbot focused on treating mental health diseases. It acts like a therapist, allowing the provision of primary psychotherapy care. Using AI, it provides a bunch of functionalities capable of aiding its user, such as calming exercises, sleeping exercises, exercises to fight/reduce anxiety, breathing exercises and also exercises to improve low self-esteem. Elomia fulfilled its goals insofar as it reduced depressive tendencies up to 28% and anxiety tendencies by up to 31%. Youper [27] is other AI-powered chatbot that aims to help combat mental health disorders, specifically anxiety and depression. It utilizes every interaction with the user to encourage positive emotions. If the chatbot detects that the user is in a state of anxiety or depression, it suggests exercises to alleviate the symptoms. Within two weeks of using the app, users reported a moderate reduction in symptoms of anxiety and depression. The app was able to maintain the decrease in anxiety symptoms over the four-week period with an effect size of 60% [26]. The decrease in depression symptoms was also sustained over the four-week period, with an effect size of 42%, although there was a slight increase in depression symptoms between weeks 2 and 4. Moreover, users of Youper reported high success in managing their negative emotions through the app’s conversational AI feature [27].

### 2.3. Cancer-Specific Chatbots

In the context of the fight against cancer, the One Remission Chatbot [15] was developed with the goal of providing individuals affected by cancer with the information they need. In English, the chatbot offers a comprehensive list of post-cancer practices, diets, and daily exercises to aid users in managing their health and reduce their dependence on doctors. For example, it allows users to search for cancer-related risks and benefits of specific foods, and it offers the option to consult a real oncologist at all times. One Remission also serves as a mental and physical health assistant, enabling patients to share thoughts and obtain accurate explanations to their questions, either verbally or through text messages, whether they need advice on diets, exercise, or sleep.

CancerChatbot [28] is another cancer-specific chatbot. It collects user data to provide relevant information and can list all the symptoms of any type of cancer upon request with the option of explaining them further if needed. The chatbot also offers information on cancer stages and treatments.

### 2.4. Dementia-Specific Chatbots

Care [29] is a chatbot that aims to enhance brain health and reduce the risk of dementia by promoting regular cognitive engagement and memory recall. It achieves this by offering a series of questionnaires on various topics, which are primarily focused on biographical information. The chatbot is available on Telegram, and some questions are designed with multiple-choice options to accommodate elderly individuals with limited technical skills. Users can select an answer from the provided choices, which will be saved as their response to the corresponding question. AlzBot [30] is a chatbot specifically designed to assist individuals with Alzheimer’s disease, which is a form of dementia. In addition to providing conversation, it also displays reminders provided by the caregiver.

### 2.5. COVID-19-Specific Chatbots

Most of the chatbots related with COVID-19 aim to accurately answer COVID-19 questions, preventing the spread of misinformation among the public. Some examples are Cosibot, COVID-19 Leave Chat Bot [31], and the MyGov Corona Helpdesk chatbot [32]. Cosibot has a Portuguese version, titled Helena, which is currently disabled. COVID-19 Leave Chat Bot only provides information about the Oregon state in the United States of America. The MyGov Corona Helpdesk chatbot also assists citizens in finding nearby COVID vaccine centers.

ANA [33] is a chatbot assistant designed for Brazilian Portuguese speakers that provides education and information on COVID-19 while also monitoring suspected cases based on patients’ symptoms and comorbidities. However, this monitoring service is currently only available for the cities of Belo Horizonte and Divinópolis in Brazil. COVIBOT [34] is another chatbot that helps users seek appropriate medical attention based on their symptoms and provides information on nearby hospitals. Lastly, ChatBot-19 Risk Assessment Chatbot [31] calculates the user’s risk of contracting COVID-19 based on information such as their symptoms, age, and gender. It is worth noting that other commonly used chatbots for diagnosis (Section 2.1), while not specifically designed for COVID-19, are still capable of diagnosing the disease based on symptoms.

## 3. *GECA* Chatbot

After our literature review, it was noticed that most existing chatbots are not focused on preventive care, more specifically for patients who are undergoing home treatment due to some specific condition. Even context-specific chatbots only cover some of the points where a chatbot can help the patient after a diagnosis. For example, cancer-specific chatbots provide information about cancer symptoms, post-cancer practices, etc.; however, they do not monitor patient symptoms and alert them to see a doctor if a specific symptom is abnormal. Similarly, when it comes to COVID-19 chatbots, as far as we know, none of them assist patients in monitoring their symptoms during the quarantine period or provide guidance on necessary actions based on medical recommendations and prescriptions. Also, dementia-specific chatbots are more focused on helping patients with reminders rather than trying to monitor their dementia status and help them fight disease progression.

**Figure 1:**
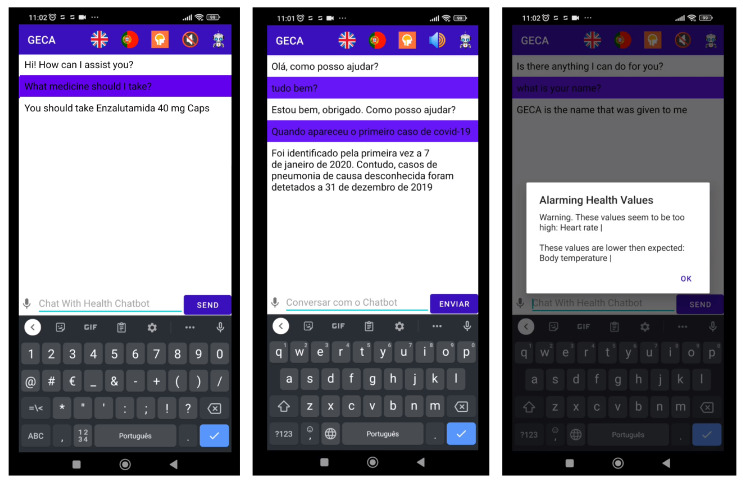
*GECA* Information and alarm screens.

To address this gap, in this work, we present *GECA*, which is a chatbot assistant for preventive care. *GECA* will not only provide medical information to the users but also monitor the progression of user’s health status at home. As a result of its integration with specific external sources, *GECA* chatbot can obtain access to a patient’s prescribed medication and health device measurements (Figure 1). This enables *GECA* to provide advice on adherence to the treatment plan and detect potential dangers if the measured parameters are at risk of exceeding the reference ranges. Based on the user’s profile, *GECA* can suggest preventative measures, which are continuously updated using data from external sources (e.g., medical devices, prescription software, etc.) and historical data collected during the user’s interactions with the chatbot.

As a first step, two health issues, COVID-19 and dementia, have been selected as areas where GECA can assist users due to their significance. The COVID-19 pandemic has highlighted the importance of remote monitoring of individuals to avoid hospitalization. *GECA* can help monitor a COVID-19 positive patient at home by providing instructions for taking medication, measuring health parameters, and monitoring symptoms. If the therapeutic plan is not being followed or if the measured parameters show a risk of exceeding the reference intervals, *GECA* offers preventive measures, such as a notification for the user to contact the doctor. Early detection of a worsening condition is crucial, and this applies not just to COVID-19 but to all illnesses. Moreover, like other COVID-19 chatbots, *GECA* also provides general information on the disease (Figure 1).

**Figure 2:**
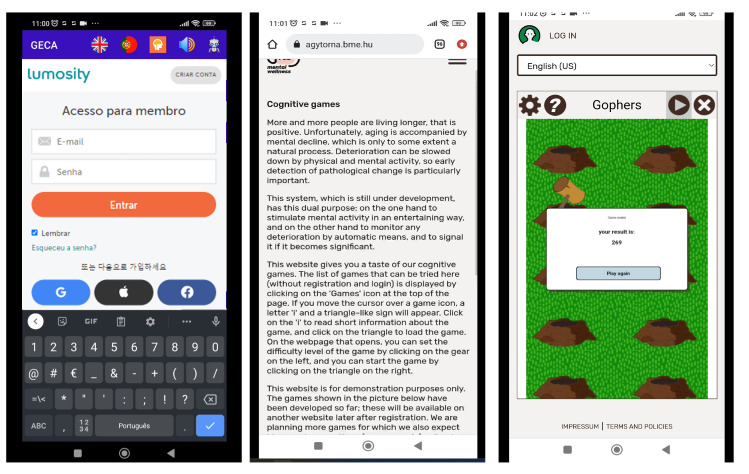
*GECA* Cognitive games screens.

*GECA* can offer monitoring services to patients with dementia in a similar manner. Since adherence to therapy is critical for managing dementia, *GECA* can be an added value for such patients. Furthermore, as monitoring the progression of the disease is essential, *GECA* offers cognitive games (Figure 2) that serve as a vital tool in managing cognitive disorders such as dementia, according to Michael and Chen [40]. By using the results of these cognitive games, *GECA* can recommend preventive measures to the user or remind them to contact their primary physician, who can utilize the results to make informed decisions about the user’s treatment.

Finally, it is worth noting that *GECA* supports interaction in both European Portuguese and English, as can be seen in Figure 1. This feature is particularly valuable, as there are few health chatbots that can understand European Portuguese. Furthermore, the ability to communicate through both voice and text makes it accessible and convenient for individuals who may have difficulty with reading or writing. This not only improves *GECA*’s accessibility but also enhances the overall user experience.

### 3.1. Architecture

Nowadays, there are several chatbot frameworks available which provide pre-built modules and tools to simplify the process of developing a chatbot [41]. Some examples are Watson from IBM, Dialogflow, LUIS, and RASA. However, using these kinds of platforms can have a huge impact on the flexibility, customization and integration of the developed chatbot. Therefore, to implement *GECA*, it was decided to use Python and Kotlin programming languages. The latest was chosen because the current version of *GECA* is available on the Android platform.

**Figure 3:**
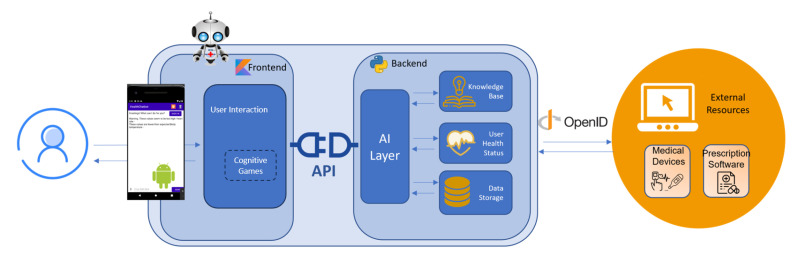
*GECA* architecture.

The overview of *GECA* architecture is depicted in Figure 3, which consists of two distinct parts: the frontend and the backend. The user interacts with *GECA* through the frontend. It is responsible for displaying the chat interface to the user and receiving their input. Using this interface, the user interacts to ask questions or provide information to *GECA*. Then, the frontend sends the user’s input to the backend for processing and displays the chatbot’s response back to the user. The cognitive games are also part of the frontend interface, since *GECA* has connections with different game providers (Figure 2), such as lumosity [42] or mental wellness [43]. The communication between the backend and frontend is ensured by an API.

*GECA* backend is responsible for processing the user’s input, interpreting it, and generating an appropriate response. This involves using NLP techniques to extract meaning from the user’s input; accessing knowledge databases, data storage and external resources to retrieve information; and generating a response that the frontend can display to the user. The AI layer serves as the orchestrator for all of these functions. Using different NLP techniques, namely tokenization and intent recognition, *GECA* processes the user message to understand the user’s input and intent to provide a relevant response. Then, *GECA* considers the message received, the knowledge already acquired and the user’s health status to send the most suitable response to the user.

The knowledge base of *GECA* is composed of various sources of information, including domain-specific knowledge to answer medical-related questions, context awareness to provide personalized and relevant responses to users over time, and NLP tools used to understand the user’s intent. Several bodies such as the World Health Organization provide domain-specific data that can be very helpful to include in the knowledge base. Multiple AI techniques are utilized to create more broader knowledge, and several models are trained using it that can be leveraged by *GECA*. For example, by utilizing models previously trained and stored in the knowledge database, *GECA* can detect various patterns in the user’s health status using data collected from different health devices, such as temperature and heart rate, and suggest calling a doctor. To ensure these models remain up to date, all gathered information, including information from external sources like medical prescription software, is stored in a data repository. This enables the acquisition of contextual information about the user and the provision of more accurate interactions, which is critical since personalization is a crucial aspect of a health chatbot.

**Figure 4:**
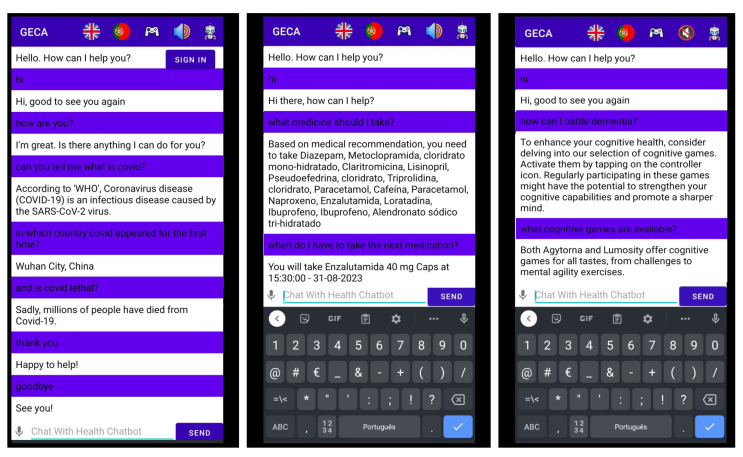
*GECA* dialogue examples.

Users can interact with *GECA* through an Android application using text and/or voice in either English or European Portuguese. To enhance user engagement, *GECA* is programmed to respond to polite messages like greetings and farewells, and it always initiates the conversation to maintain proactive communication. Additionally, to keep the conversation going, *GECA* sends periodic messages while also remaining focused on proactivity and engagement. While *GECA* does not provide off-topic health advice, it does offer generic responses to such queries. If *GECA* fails to understand an input, it prompts the user to rephrase their statement. The app also includes symbols such as flags representing the UK and Portugal to enable language selection and a visually appealing *GECA* avatar to facilitate a more interactive and emotionally connected conversation with users. Figure 4 displays different examples of dialogues involving *GECA* and the user.

### 3.2. Implementation

Understanding the user’s input is a crucial aspect of any chatbot implementation. In the development of *GECA*, the Natural Language ToolKit (NLTK) Python library was chosen as the primary tool for this task. This widely used natural language processing library simplifies working with natural language in Python programs. It includes a range of useful libraries for tokenization and stemming as well as model training. Tokenization involves breaking strings into smaller substrings that can be used to identify semantic similarities between user input and the intent database’s patterns. This helps identify the most appropriate question–answer set. Stemming, on the other hand, produces morphological variations of a word to find the best match between input phrases and pattern files.

Since *GECA* is a domain-specific chatbot, correctly interpreting user intent is essential to understanding what is expected from the conversation. This is achieved through intent classification, which enables *GECA* to provide the correct response in each interaction. By using neural networks and the functionalities provided by the NLTK Python and Tensorflow libraries, GECA can provide consistent replies during the dialogue. The AI layer is responsible for this process.

Another important concern during *GECA* implementation was the security and standardization of the data. Since *GECA* interacts with external resources that share sensible information about the user, namely medical prescriptions and measurements provided by health devices, the security of the data is of paramount importance. Therefore, it was decided to use OpenID protocol that reduces the risk of password-related vulnerabilities, providing centralized authentication, giving users control over their personal data, and ensuring a standardized protocol for authentication and authorization [44]. This is a distinguishing feature of *GECA* because, even though there is a potential risk of hacking or data breaches that could compromise the confidentiality of sensitive health-related information, most existing chatbots do not prioritize security as a critical factor [45].

**Figure 5:**
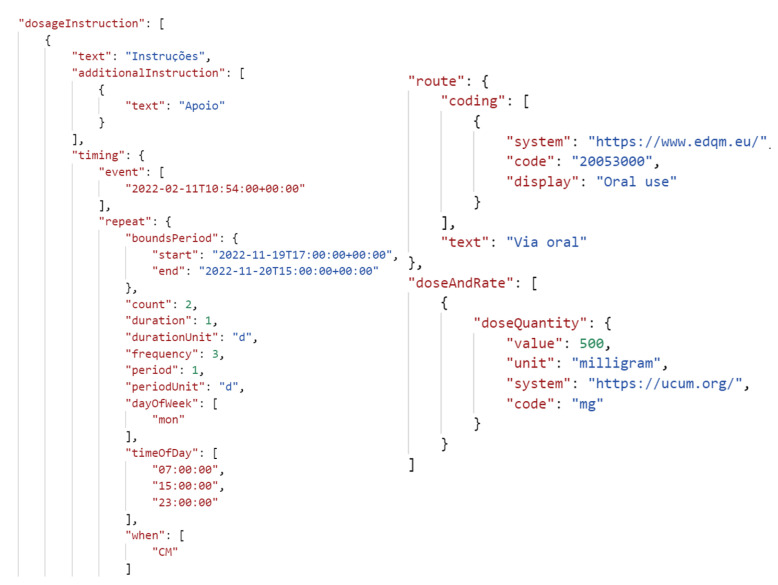
FHIR excerpt example: medication prescription.

To ensure standardization, *GECA* utilizes the Fast Healthcare Interoperability Resources (FHIR) standard to obtain all external healthcare information. FHIR is a modern web-based healthcare standard that enables the seamless exchange of healthcare data electronically, and it is easily adoptable by diverse organizations, such as hospitals, laboratories, and insurance companies [46]. By leveraging FHIR, *GECA* can efficiently share healthcare data across various sources, while its modularity and extensibility allow for the incorporation of different healthcare data types and adaptation to evolving use cases. Figure 5 showcases an excerpt of an FHIR file that communicates comprehensive information about a prescribed medicine to the user. Specifically, it encompasses details regarding the prescription date, format, quantity, dosage, along with instructions concerning aspects such as timing, duration, and frequency for the user’s medication intake.

The communication of *GECA* with external sources of data is completed through an API to ensure the secure transmission of FHIR files for clinical data and medication information. This API, developed using Flask, enables smooth interaction between external applications responsible for data transmission and the chatbot itself. Through this API, *GECA* gains access to the user’s medical data, such as clinical values and prescribed medications, while maintaining the utmost privacy and security of the information. Consequently, *GECA* can promptly notify the patient if any abnormalities are detected in their health values or provide essential information about their prescribed medications.

**Figure 6:**
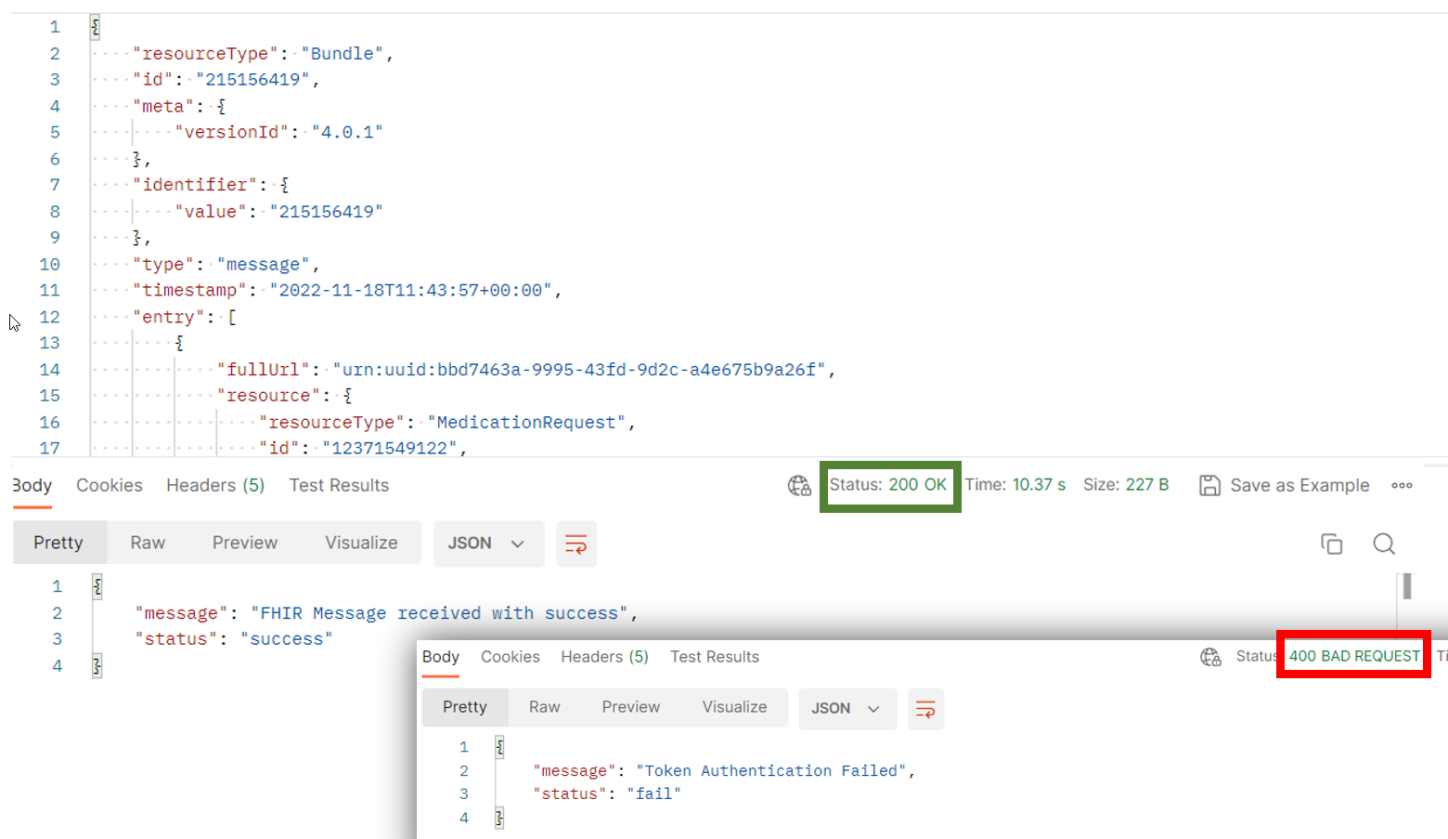
*GECA* API request example.

To establish a secure and controlled access to the API, a token-based authentication mechanism, specifically OAuth 2.0, is used. By implementing OAuth 2.0, the API ensures secure communication, protecting sensitive medical data from unauthorized access and maintaining a high level of data privacy for patients. Therefore, when external applications or users attempt to interact with the API, they must first obtain an access token by authenticating themselves through OAuth 2.0. These access tokens are time-bound and grant limited permissions, ensuring that only authorized entities can access specific resources within the API. The usage of short-lived access tokens reduces the risk of unauthorized data exposure and unauthorized access. In addition to facilitating communication, error handling, and validation, the API provides specific status codes as responses to incoming FHIR format data. Upon the successful transmission of valid information, the API promptly issues a 200 status code, as illustrated in Figure 6. Conversely, when the format is incorrect or the medical data lack an authentication token, the API responds with a 400 status code, indicating the presence of an error and providing relevant details regarding the encountered issue (Figure 6). This approach ensures a secure yet streamlined communication channel between external entities and *GECA*.

### 3.3. Validation

Currently, standardized protocols for chatbot evaluation have not been universally established. Consequently, each developer tends to adopt their own unique approach, frequently relying on empirical experimentation. Furthermore, given the critical nature of this domain, comprehensive assessments must encompass a spectrum of factors, including usability, social interaction quality, response accuracy, and, where relevant, classifier performance. Hence, it becomes imperative to consider the formulation of guidelines specifically tailored for health chatbot evaluators, delineating the essential metrics, and delineating when and how to conduct evaluations during the product development lifecycle.

During this phase of *GECA*’s development, it becomes imperative to execute functional testing, aiming to affirm the seamless operation of *GECA*’s fundamental features. This entails scrutinizing elementary interactions to ascertain its proficiency in comprehending and responding to user inputs as intended. Equally crucial is the validation of the accuracy and pertinence of *GECA*’s responses. Additionally, data security assumes paramount importance, necessitating meticulous attention to ensure that *GECA* adeptly manages user data in accordance with pertinent security and privacy regulations.

Two distinct scenarios were devised to validate the capabilities of *GECA*. Initially, to evaluate GECA’s ability to provide accurate responses, a test set consisting of 400 messages in both Portuguese and English was developed. Most of the questions pertained to COVID, but a few out-of-scope messages were also included. This dataset served as a means to scrutinize the quality and appropriateness of *GECA*’s conversational responses. After the testing, *GECA* achieved a 97% success rate in providing correct answers with the remaining 3% of incorrect responses attributed to out-of-scope questions. These results are very good and consistent with the existing literature, which suggests that a well-designed and trained chatbot should be able to answer at least 90% of inquiries posed [47].

An additional scenario was constructed to assess *GECA*’s capacity to oversee patients’ health statuses. To achieve this, a dataset containing 10 FHIR files, each containing diverse patient data, was assembled. These files were subsequently transmitted to *GECA* through the same API employed by external sources for communication. This approach allowed us to comprehensively evaluate not only *GECA*’s proficiency in monitoring patient conditions but also the effectiveness of the entire communication pipeline. As a result, *GECA* successfully processed all the data as anticipated, promptly notifying patients when values fell outside the established normal ranges. By adhering to established standards and implementing the OpenID protocol, compliance with security and privacy regulations is ensured.

In summary, based on the conducted tests, *GECA* has demonstrated its accurate and precise functionality when engaging with the various suggested features within the two scenarios. Furthermore, the testing experiments have underscored the usability and effectiveness of *GECA*’s comprehensive functionalities. However, it is important to acknowledge that these initial validations represent just the beginning. A more comprehensive evaluation, encompassing a diverse user base and considering measurements across various dimensions, is warranted. While factors such as usability, social interaction quality, and performance remain central in chatbot evaluation, the overall quality in real-world usage holds paramount significance. Therefore, this will be the next step in our research, involving a broader spectrum of users and encompassing an array of dimensions for assessment. While the accuracy and usability of *GECA*’s functionalities have been established in these initial validations, the next evaluation phase will provide a more holistic understanding of its performance in real-world scenarios.

## 4. Conclusions

In the healthcare sector, several chatbots have been developed for various purposes, but there is a lack of focus on preventive care for patients undergoing home treatment for specific conditions. To bridge this gap, *GECA*, a preventive care chatbot has been developed to furnish patients undergoing home treatment with information, advice, and monitoring. Currently, *GECA* is targeted toward patients with COVID-19 and dementia, providing guidance on medication, health conditions, and monitoring symptoms. However, the capabilities of *GECA* can be extended to other diseases and conditions with ease. Moreover, *GECA* is programmed to notify users to seek medical attention if any abnormal behavior is detected. Apart from its home health management features, *GECA* also provides standard health information, including COVID-19 related details, to users. In addition, cognitive games are offered as a means to combat cognitive diseases, particularly for patients with dementia.

The availability of text and/or voice interaction in both European Portuguese and English on *GECA* makes it more accessible and convenient for users, including those with difficulties in reading or writing. This enhanced accessibility also contributes to a better user experience. The platform’s ability to connect with external resources is another advantage, as it allows for a higher degree of personalization, which is a critical factor in user engagement. The use of FHIR standards in these connections facilitates easy adaptation to new healthcare information sources. To ensure user security, all communications on *GECA* utilize the OpenID protocol. The implementation of these standardized practices and robust security protocols can foster increased confidence among healthcare providers and policymakers when promoting patients to make use of *GECA*.

*GECA* has demonstrated a remarkable level of accuracy and precision in its interactions with the diverse features, boasting an impressive 97% success rate in delivering accurate responses. Furthermore, the testing experiments have emphasized the usability and efficacy of *GECA*’s extensive capabilities. Nevertheless, a wider range of factors should be analyzed to ensure the chatbot’s success in real-world scenarios. This involves considering guidelines explicitly tailored for health chatbots, adhering to vital metrics. Therefore, as part of future work, we plan to test and evaluate the effectiveness, efficiency, and goal achievability of *GECA* as well as its ability to satisfy users. While user satisfaction is a common metric used to evaluate chatbots, these other categories are also important. Quality is another pivotal metric given the paramount importance of adhering to quality standards such as ISO/IEC 25010 in the healthcare domain [48]. This will be a challenging undertaking, but the guidelines provided by Maroengsit et al. [1] will be used to select appropriate evaluation methods for assessing *GECA*. As an integral component of the iCare4NextG platform, *GECA* has been smoothly incorporated into a widely used Portuguese software employed in the majority of Portuguese hospitals. Currently, a pilot initiative is underway, in collaboration with a Portuguese hospital, healthcare providers and policy makers, aiming to evaluate and assess the effectiveness and impact of *GECA* within this specific context.

## Figures and Tables

**Table 1:** Selected chatbots summary.

Chatbot Purpose	Chatbot Name
Diagnosis	Babylon [15]
	Ada Health [16]
	Buoy Health [17]
	Your.md [18]
	Gyant [19]
	Symptomate [20]
	MedWhat [21]
Well-Being	Iona Mind [22]
	Florence Nightingale Chatbot [23]
	Izzy [24]
	SafeDrugBot [25]
Mental Health Chatbots	Elomia [26]
	Youper [27]
Cancer-Specific Chatbots	One Remission Chatbot [15]
	CancerChatbot [28]
Dementia-Specific Chatbots	Care [29]
	AlzBot [30]
COVID-19-Specific Chatbots	COVID-19 Leave Chat Bot [31]
	MyGov Corona Helpdesk chatbot [32]
	Cosibot [31]
	ANA [33]
	COVIBOT [34]
	ChatBot-19 Risk Assessment Chatbot [31]

## Data Availability

Data sharing not applicable.
